# Policy and Governance Perspectives for Regulation of Genome Edited Crops in the United States

**DOI:** 10.3389/fpls.2018.01606

**Published:** 2018-11-08

**Authors:** Jeffrey D. Wolt, Clark Wolf

**Affiliations:** ^1^Department of Agronomy, Crop Bioengineering Center, Iowa State University, Ames, IA, United States; ^2^Department of Philosophy, Department of Political Science and Bioethics Program, Iowa State University, Ames, IA, United States

**Keywords:** jurisprudence, administrative law, CRISPR, Coordinated Framework, GMO

## Abstract

Genome editing for crop improvement lies at the leading edge of disruptive bioengineering technologies that will challenge existing regulatory paradigms for products of biotechnology and which will elicit widespread public interest. Regulation of products of biotechnology through the US Coordinated Framework for Biotechnology is predicated on requiring burden of proof that regulation is warranted. Although driven by considerations of newly emerging processes for product development, regulation has, for the most part, focused on characteristics of the biotechnology product itself and not the process used for its development *per se*. This standard of evidence and product focus has been maintained to date in regulatory considerations of genome edited crops. Those genome edited crops lacking recombinant DNA (rDNA) in the product intended for environmental release, lacking plant pest or pesticidal activity, or showing no food safety attributes different from those of traditionally bred crops are not deemed subject to regulatory evaluation. Regardless, societal uncertainties regarding genome editing are leading regulators to seek ways whereby these uncertainties may be addressed through redefinition of those products of biotechnology that may be subject to regulatory assessments. Within US law prior statutory history, language and regulatory action have significant influence on decision making; therefore, the administrative law and jurisprudence underlying the current Coordinated Framework strongly inform policy and governance when considering new plant breeding technologies such as genome editing.

## Introduction

Society now faces a wave of disruptive biotechnology innovation extending from uses of DNA as an information storage medium to applications of human genome editing and synthetic biology (NASEM, 2017). The use of genome editing for crop improvement is at the crest of this wave. Public uncertainty surrounds genome editing and its uses (O’Keefe et al., 2015), even though the scientific underpinnings for the genome editing of plants extend to the last century (Songstad et al., 2017) and regulators have been evaluating plants modified through genome editing since at least 2004 (Camacho et al., 2014).

Declining societal trust in emerging technology predates applications of modern biotechnology, but public resistance to genetic modification as tampering with nature stands as a particularly strong example of how public attitudes toward new technologies have been at odds with scientific institutions, regulatory authorities and traditional information providers (Frewer, 1999). Increasing skepticism of plant biotechnology is evident in the US. The first commercial uses in 1994 were met perhaps with more public curiosity than concern (Bruening and Lyons, 2000), but there has been a steady decline in public support to the point where in 2015, only 37% of the public viewed genetically engineered (GE) foods as safe as compared to 88% of scientists from a wide range of disciplines (Funk and Rainie, 2015).

Against this backdrop, regulators in the US have found no basis in existing regulation to encumber potential entry of genome edited crops into commercial use when the intended product shows no evidence for presence of recombinant DNA (rDNA) (Wolt et al., 2016). But in recognition of the massive amount of product innovation that may arise from emerging genome engineering technologies, including genome editing, there is increasing focus on the role of scientific and public governance mechanisms for decision making regarding future products of biotechnology (NASEM, 2017). Here we focus on bioengineering of plants and consider the historical interactions of regulatory policy and extra-regulatory governance mechanisms as they relate to decision making regarding GE crops. Further, we consider the implications of policy and governance for the emergence of genome edited crops and their derived products. While governance may be considered in a wide variety of contexts, we focus on concepts of jurisprudence applied to rule of law which reflects the administrative governmental structure of the United States (Stack, 2015).

In this paper, we begin with a review of the existing regulatory regime covering biotechnology-derived plants in the United States. Since the regulatory environment changes over time, we include mention of some of the important events that have shaped the present regulatory environment. Existing regulations are vague and ambiguous in their application to new technology, especially genome edited crops. While it might seem obvious that genome editing is biotechnology, genome edited crops need not contain genetic material from other organisms, and might contain no new DNA material at all – in some cases, editing simply involves removing or disabling a bounded genetic sequence or set of sequences. Regulators and others who wish to interpret existing and pending statutes and to understand its implications are therefore faced with a quandary. It is not obvious, *a priori*, to include genome edited organisms under existing regulations covering GE products, or as “products of biotechnology,” a term with shifting meaning as applied in law (Executive Office of the President [EOP], 2016). The problem is one of legal interpretation in the context of regulatory decision making. Accordingly, the second part of the paper addresses the problem as a question of jurisprudence, considering alternative theories of legal interpretation from the perspective of administrative law in the effort to evaluate their implications for genome edited foods and crops. The review and analysis in this paper particularly addresses regulation in the United States. Our goal is to explain and evaluate aspects of the *status quo* in US regulatory law as it impinges on accommodating genome edited crops within the Coordinated Framework for Biotechnology.

## Emergence of the Us Governance Frameworks for Biotechnology

Scientific realization of the potential and implications of rDNA methods led in the 1970s and 1980s to widespread discourse within the scientific community and federal agencies as to the need for oversight specific to both the processes and products of biotechnology (National Research Council [NRC], 1989). Early discussions focused on the science were broadened to encompass ethical issues and legal liabilities. This culminated in the call from the Asilomar Conference for stringent scientific self-governance until the broader safety implications of rDNA technology could be understood (Berg et al., 1975).

The National Institutes of Health (NIH) formalized, through the Recombinant DNA Advisory Committee (RAC), the statement of principles coming from the Asilomar Conference in the form of binding guidance for contained use in NIH-funded research (US National Institutes of Health [NIH], 1976). This guidance was subsequently relaxed in light of improved understanding of the risks associated with the technology (US National Institutes of Health [NIH], 1978). Throughout the late 1970s and the early 1980s, NIH guidelines evolved to decentralize administration, reduce duplicative review processes, exempt certain types of experiments from review and to broaden scope of the guidance for considerations of human gene therapy and environmental releases (National Research Council [NRC], 1989). The NIH guidelines were adopted throughout federal agencies, and they influenced thinking and actions surrounding rDNA research and development in the private sector.

Influenced in part by *Diamond v. Chakrabarty*, a court challenge that upheld patentability of life forms (United States and Supreme Court, 1980), and thus encouraged commercial development in biotechnology, Congressional hearings considered the adequacy of oversight mechanisms for GE organisms. These hearings concluded that existing statutory mechanisms were adequate to govern the technology but could benefit from clarification. Further, because there was, at the time, no way to quantify the risks posed by GE organisms to the environment, federal agencies were not able to assess risks to the environment for purposes of regulation (National Research Council [NRC], 1989). Concurrent with these Congressional oversight hearings, the White House Cabinet Council on Natural Resources and the Environment formed an interagency working group which initiated the process leading to formal coordination of biotechnology oversight activities among federal agencies (National Research Council [NRC], 1989).

## The Us Coordinated Framework for Biotechnology

Beginning in 1984, a series of interagency working groups began in-depth evaluation of applicable laws for oversight of biotechnology, and the agencies most active in addressing biotechnology began formalizing their regulatory roles and policies. Following a shift in biotechnology coordination to the Office of Science and Technology Policy (OSTP), that office released the Coordinated Framework for Regulation of Biotechnology establishing regulatory responsibilities, lead agencies and jurisdictions relying on existing laws for oversight of biotechnology (Office of Science and Technology Policy [OSTP], 1986). As reflected in the Coordinated Framework, “the overall thrust of the regulatory response to biotechnology may be termed a minimalist, cost-effective, priority-driven approach requiring burden of proof that regulation is warranted,” (Krimsky and Wrubel, 1996).

The most consequential regulatory approach to emerge from the Coordinated Framework was a shift away from earlier oversight considerations based on the biotechnology process used and toward the product of bioengineering. A critical consideration at the time was whether classical mutagenesis would be caught in the snare of product-focused assessments to force regulatory oversight of products which had not traditionally been subject to regulation (National Research Council [NRC], 1989). This concern stands in juxtaposition to current-day considerations of products developed through site-directed mutagenesis via genome editing where there are questions as to whether these products are analogous to products of classical mutagenesis, which remain outside of regulatory purview, or whether they are uniquely products of biotechnology that are to be regarded within the existing regulatory frameworks in the US and elsewhere (Sprink et al., 2016; Wolt et al., 2016).

The Coordinated Framework has been mutable over time, changing in response to advances in biotechnology innovation, knowledge gain, improved understanding of risks and uncertainties, and changing appreciation of the technology. This has been accomplished without new or revised legal statutes, but rather through the less onerous process of regulatory rulemaking and changes in regulatory guidelines.

In 1992, the Coordinated Framework was updated to clarify how regulatory authority should be exercised where there is latitude as to the discretion that may be taken by the implementing agency (Office of Science and Technology Policy [OSTP], 1992). The very specific language of this update to the framework emphasized that regulations should address only those risks that are “real and significant rather than hypothetical or remote” and show evidence risk is unreasonable. The policy’s emphasis on health and safety has been construed by some as excluding considerations of societal impacts leading to “few meaningful opportunities for citizens to consider either the nature of the risks or their acceptability in the larger social context of the potential harms,” (Kelso, 2003), however, obligations under the National Environmental Policy Act (NEPA) (Congress, 1969) mandate actions taken under the Coordinated Framework consider the effect on the human environment when “economic or social and natural or physical environmental effects are interrelated” (Code of Federal Regulations [CFR], 2003). Further, Congress has specified that policy, regulations and laws “utilize a systematic, interdisciplinary approach which will insure the integrated use of the natural and social sciences and the environmental design arts in planning and in decision making which may have an impact on man’s environment” (42 USC part 4332, United States Code [USC], 2008).

### Agency Regulatory Guidance and Rulemaking Actions

From the time of the 1992 update until later efforts to update the regulatory system for biotechnology products, beginning in 2015 (Executive Office of the President [EOP], 2015), there were no major changes brought forward to alter overarching goals and approaches outlined in the Coordinated Framework. In the intervening years, however, changes or attempts to change regulation of biotechnology were witnessed within regulatory guidance or rulemaking.

#### Food and Drug Administration

In 1992 the Food and Drug Administration (FDA) issued a policy statement on foods derived from plants developed by rDNA techniques. Further, FDA clarified their product-focused position that these foods were substantially equivalent to foods already in commerce and with the exception of “those cases when the objective characteristics of the substance raise questions of safety sufficient to warrant formal premarket review,” no explicit regulatory action was needed within FDA (US Food and Drug Administration [FDA], 1992). In 2001 the FDA issued a proposed rule to require that developers submit a scientific and regulatory assessment of a bioengineered food before it is marketed (US Food and Drug Administration [FDA], 2001). Action on this proposed rule has not been taken and FDA continues to adhere to its voluntary consultation process for foods developed with rDNA. Over the years FDA has provided guidance to industry regarding their consultation procedures, early food safety evaluation and voluntary labeling standards for foods derived from GE plants (US Food and Drug Administration [FDA], 2018a).

#### Environmental Protection Agency

The US Environmental Protection Agency (EPA) has wide-ranging authority of direct or indirect bearing on products of biotechnology. The principle statutes under which EPA considers environmental safety and human health with respect to GE crops are the Federal Insecticide, Fungicide and Rodenticide Act (FIFRA), the Toxic Substances Control Act (TSCA), and the Federal Food, Drug and Cosmetics Act (FFDCA). Crops which are GE to express plant incorporated protectants (PIPs) are considered with regard to the pesticidal protein and not the modified plant *per se*. Examples of PIPs include proteins from *Bacillus thuringiensis* (Bt), which confer insect resistance, and viral coat proteins which confer disease resistance. In addition to PIPs, the EPA indirectly considers crops GE to confer herbicide resistance (e.g., glyphosate resistance) by evaluating exposure to the herbicide (e.g., glyphosate) used to manage the crop. Over time the EPA has defined and refined their processes through specific regulatory actions (US Environmental Protection Agency [EPA], 2018).

#### Department of Agriculture

The United States Department of Agriculture (USDA), under provisions of the Plant Protection Act (PPA), provides the regulatory oversight of GE organisms to protect plant health. It does so by regulating the introduction of those GE organisms that may pose a pest risk to plants (CFR 7 part 340, Code of Federal Regulations [CFR], 1987). Beginning 2008, USDA undertook an effort to institute new rulemaking for GE organisms to encompass provisions of the Noxious Weed Act of 1972 in addition to the PPA with the intent to broaden the basis for regulation and to streamline the process for determinations of regulatory status of certain GE organisms (United States Department of Agriculture [USDA], 2008). Following extensive public comment, this proposal was withdrawn in 2015 allowing USDA to engage in new stakeholder engagement on APHIS biotechnology regulations and to initiate, for purposes of rulemaking, a programmatic environmental impact statement (EIS) (United States Department of Agriculture [USDA], 2016a,b).

#### Overarching Regulatory Authority

Agencies working within the Coordinated Framework must also address regulatory processes and determinations for products of biotechnology through federal statutes which have overarching authority. A feature of these overarching authorities is the ability for broader public involvement than is typically experienced under the Coordinated Framework.

The National Environmental Protection Act (NEPA) of 1969, as amended, requires that federal agencies take a “hard look” at how a regulatory action may affect the human environment (Department of the Interior, 2004). Under NEPA, significant environmental impacts of an action must be disclosed to the public prior to the action being taken; but the act does not dictate the nature of action to be taken based on the analysis that is performed (Bean, 2009). The agency prepares an environmental assessment (EA) and if the threshold determination is a Finding of No Significant Impact (FONSI) there is no need for further analysis. If, however, the provisional determination by the agency is that the proposed action may significantly affect the human environment, an EIS is necessary. The EIS outlines the proposed action and alternatives, and evaluates the environmental impact of each in arriving at a final action. The Council on Environmental Quality (CEQ) determines the process and need for NEPA to be applied within federal agencies. For instance, decision-making activities undertaken by EPA are considered “functionally equivalent” to those of NEPA and, therefore, there is no need to undertake an EIS. Legal challenges to how NEPA is applied to USDA petitions for nonregulated status have influenced decision making within USDA and are partially responsible for strengthening of assessments for GE crops (Cowan and Alexander, 2012).

The Endangered Species Act (ESA) (16 USC part 35, United States Code [USC], 2012) requires that federal agencies consider both direct and indirect effects of actions they take on endangered species and their critical habitat. The ESA is administered by the Fish and Wildlife Service (FWS) and the National Marine Fisheries Service (NMFS). Agencies conduct their own internal assessments regarding endangered species and if they determine “no effect,” no further action is needed. In cases where there may be an effect the agency must consult with FWS and/or NMFS to determine if the effect is “likely.” If a determination of a “likely” effect is made, then assessment responsibilities shift to FWS/NMFS where a determination is made whether the organism or habitat will be placed in jeopardy. Federal statutes applied under the Coordinated Framework have not led to any findings of likely effect for GE crops and, therefore, formal interagency consultation has never taken place (NASEM, 2017). The FDA has been sued over their obligations under the ESA with respect to transgenic AquAdvantage salmon, even though FWS/NMFS were informed of, and concurred with, FDA’s finding of no effect (Center for Veterinary Medicine [CVM], 2012). A problematic aspect of the ESA process for products of biotechnology is that assessments for GE organisms under the Coordinated Framework allow some reasonable degree of risk (see for example, Peterson et al., 2006), whereas ESA determinations are concerned with loss of a single individual. Efforts to bridge the endangered species assessment approaches used across agencies have been made (National Research Council [NRC], 2013), but have not yet been applied to bioengineered organisms.

## Governance and the Coordinated Framework for Biotechnology

Beyond direct regulatory oversight for products of biotechnology, the broader governance of biotechnology in the United States is evidenced through public comment with respect to proposed regulatory actions, formally constituted advisory committees to federal agencies, legal challenges of regulatory actions and wide-ranging civil discourse.

When new administrative regulations (rules) are proposed or when these rules are subject to change, US government agencies undertake public comment periods as stipulated under the Administrative Procedures Act of 1946 (5 CFR 553, 2012). Following an advise and consent procedure, any proposed regulation is published in the *Federal Register* and public input is solicited for a minimum of 30 days as written submissions, frequently as digital electronic submissions, and occasionally through public meetings. Before a rule is finalized the responsible agency must respond to the public record which may consist of public comment, expert opinion, scientific data and other factual evidence. Under the Coordinated Framework, rulemaking and other broad policy decisions largely encompass environmental issues and therefore public comment is addressed through programmatic reviews (a NEPA EIS) as a means to determine if the responsible agency has established the relevant baseline for the assessment (Council on Environmental Quality [CEQ], 2014). Public comment impacts the pace and nature of regulatory decisions, as for instance the determination of USDA to withdraw and rewrite the proposed rule of 2008 in response to more than 88,300 comments that addressed the scope and meaning of the rule (United States Department of Agriculture [USDA], 2016a).

When USDA assesses a GE crop through petitions for determination of nonregulated status, the EA is a more commonly used mechanism than is an EIS. The EA has a lower standard for transparency and public engagement than does the EIS, and USDA has been legally challenged to undertake NEPA EIS before granting nonregulated status (Cowan and Alexander, 2012). From 2007 through 2011, the USDA EA process and regulatory determination for nonregulated status of glyphosate resistant alfalfa, as well as conditions for the conduct and determinations arising from a court-mandated EIS, was argued through to the Supreme Court on the basis of economic considerations to growers and to export markets. As a consequence, USDA conducted an EIS, which received 135,000 public comments; the product was fully deregulated in 2011. Somewhat similar arguments, court actions and USDA responses were taken with respect to glyphosate resistant sugarbeet. Following a court-mandated EIS, the product was partially deregulated and full deregulation was undertaken under a subsequent EA. These and other challenges have led USDA to take a more formal and transparent approach to its assessments and to show a greater willingness to conduct comprehensive EIS for determinations of deregulated status.

Agencies working within the Coordinated Framework utilize advisory groups for advice and direction. As previously described, the NIH RAC is a long-standing advisory group providing direction as to procedures for federally-supported biotechnology research activity. The EPA Science Advisory Panel (SAP) meets publicly and solicits public comment in their deliberations. The SAP has undertaken numerous risk assessments and resistance management plans for Bt crops as well as considered appropriate problem formulation and testing for PIPs (NASEM, 2017). The SAP had pivotal roles in considering highly contentious issues relating to Bt maize impact on monarch butterfly and the allergenicity of food derived from Cry9C maize (Science Advisory Panel [SAP], 2000a,b, 2001). Agencies also convene expert advisory panels on an *ad hoc* basis to address issues relevant to assessing on-going programs and to providing direction as to regulation of emerging biotechnology. Oftentimes committees convened through the National Academies of Science, Engineering and Medicine (NASEM) are empaneled to consider issues relevant to biotechnology and its regulation, as for instance, recent activities to consider GE crops, gene drive technology and the future regulatory landscape for products of biotechnology (NASEM, 2016a,b, 2017).

Along with formal processes of deliberation and regulatory decision making, wide-ranging social discourse on GE crops contributes to the broader governance of the technology, but may hinder effective governance as well. Increased regulatory scrutiny over time as evidenced in increased study requirements, higher development costs and longer decision-making timelines can be ascribed in part to pressure brought about through public groups questioning and challenging the regulatory process (Smyth et al., 2014). This increased scrutiny of GE crops has also engendered extra-regulatory governance activity meant to inform regulatory process through transparent public engagement. An example is the Pew Initiative on Food and Biotechnology which from 2000 to 2007, examined and reported far-ranging issues of genetic modification of foods and the ability of the federal government to assess GE-derived food risks and benefits (Pew Trust, 2007). The commercial advent of GE crops is contemporaneous to the development of the internet, and the internet has been pivotal in dissemination of views on GE crops (Wunderlich and Gatto, 2015), however, rather than strengthening technology governance, internet communication has served to polarize positions, especially with the rise of social media where like-minded opinions become reinforced (Smith et al., 2013). Unsurprisingly, evidence is accruing that social media has been used to purposely sow dissenting positions concerning GE crops in the United States (Dorius and Lawrence-Dill, 2018).

## Current Regulation and Governance of Genome Edited Crops

Although regulation under the Coordinated Framework is frequently described as product focused, the regulatory approach to GE organisms in fact reflects a *de facto* process-based trigger in many instances (Wolt, 2017). Regulation by USDA, for example, has in the past been considered mostly when using *Agrobacterium tumefaciens*, a plant pest for the introduction of rDNA. Genetic engineering to produce the same product with rDNA introduced using biolisitcs, does not meet this standard; thus, it is possible for identical products to be evaluated differently because of the process involved. Similarly, herbicide tolerance arising naturally through spontaneous mutation (e.g., Kidwell et al., 2015) is not subject to regulation, but using genetic engineering to accomplish the same would be of regulatory concern. The EPA follows a more product-focused approach because it restricts its considerations to pesticidal products, and FDA has held firm to the idea that characteristics of the food product are the relevant regulatory concern.

The regulatory conundrum regarding process versus product poses increasing uncertainty with the advent of genome editing. Genome editing can result in a host of outcomes extending from point mutations to safe harbor transgene insertions (Wolt et al., 2016). While products comprising transgene insertions clearly fall within the regulatory realm of GE crops, the products of simple point mutations may be absent of rDNA and may represent genotypes and phenotypes that are indistinguishable from plant variation which may arise in nature. A case in point is sulfonylurea tolerant canola developed by oligonucleotide mediated mutation (Sauer et al., 2016), a form of genome editing; this was first developed in the late 1990s and has been subject to regulatory considerations worldwide since 2004 (Camacho et al., 2014; Wolt et al., 2016). The same trait has been achieved using conventional mutagenesis (Tonnemaker et al., 1992), which is not subject to regulation throughout most of the world. In Canada and the United States, the genome edited product has entered the marketplace, but its regulatory fate remains uncertain in the EU (Sprink et al., 2016).

The USDA has been the most active US agency in dealing with genome edited crops and responded to the first inquiries regarding these crops as early as 2004 (Camacho et al., 2014). The process that enables inquiries is the “Am I Regulated?” portal where USDA accepts Regulated Articles Letters of Inquiry regarding the potential for proposed products of biotechnology to be subject to regulation (United States Department of Agriculture [USDA], 2017c). In responding to these inquires, USDA has not viewed genome edited crops as subject to regulation when the edit involves simple insertion/deletions of limited numbers of bases and the absence of rDNA in the finished product (Wolt et al., 2016). Thus, for instance, herbicide resistance developed through single nucleotide substitutions, which can arise as spontaneous mutation or through conventional mutagenesis (Kidwell et al., 2015; Rizwan et al., 2015), is not subject to regulation by USDA. Similarly, the aforementioned use of genome editing to develop herbicide resistant canola is not subject to regulation by USDA. However, in situations where small native template or directed transgene insertions occur, there remains regulatory interest (Camacho et al., 2014). Thus, a case-by-case paradigm drives regulatory considerations of genome edited crops, but consistency in actions by USDA provides developers with an operational roadmap.

Beginning 2015, the Executive Office of the President (EOP) initiated an activity to update the Coordinated Framework for Biotechnology to clarify regulatory responsibilities and to assure the ability to deal with future products of biotechnology (Executive Office of the President [EOP], 2015). As defined by the EOP, biotechnology products “refers to products developed through genetic engineering or the targeted or *in vitro* manipulation of genetic information of organisms including… some of the products produced” by these organisms (Executive Office of the President [EOP], 2016). Concurrently, efforts were initiated by USDA to broaden their remit for assessing products of biotechnology and by FDA to better understand the ways that foods derived from genome edited plants may differ from conventionally derived foods in terms of food safety.

The 2016 USDA programmatic EIS announced the intent of USDA to undertake new rulemaking for GE organisms and the various options under consideration (United States Department of Agriculture [USDA], 2016b). And in early 2017, the agency announced proposed actions to update regulatory oversight for biotechnology (United States Department of Agriculture [USDA], 2017a). Exceptions were made to explicitly exclude conventionally- and mutagenically-derived organisms. Further, the distinction of product versus process as a regulatory trigger was complicated through a redefinition of genetic engineering to “mean techniques that use recombinant or synthetic nucleic acids with the intent to create or alter a genome,” thus signaling the focus on *use* of a defined process as the trigger for regulatory considerations. Exceptions to this definition involved processes of directed genome altering (i.e., genome editing) resulting in deletion of any size DNA segment, or occurrence of a single base pair substitution that could otherwise result from the use of chemical- or radiation-based mutagenesis, or genome editing-enabled insertion of DNA segments that could have been achieved through traditional breeding with a sexually compatible species. Further, null segregant progeny of a GE organism could be excluded from regulation when the rDNA or synDNA inserted into the recipient genome was not passed to the recipient progeny and there was no alteration of the DNA sequence of the progeny (United States Department of Agriculture [USDA], 2017a). These exclusions and further language in the proposed rule would make distinction amongst the means of genome editing similar to that currently reflected in USDA actions in response to Regulated Article Letters of Inquiry for genome edited crops. That is, a determination as to whether the product would be subject to regulatory consideration would be based on whether the modification within the progeny’s genome involved deletions, point insertions, or native template insertions, and whether rDNA or synDNA remained in the modified organism (see for instance, Wolt et al., 2016). In addition to these process/product definitions, the proposed rule invoked both plant pest and noxious weed considerations to provide greater statutory support for USDA’s regulations, an approach which proved problematic in USDA’s earlier attempt at rulemaking (United States Department of Agriculture [USDA], 2008, 2016a). Based on public comments expressing a wide range of concerns regarding the new proposed rule, USDA has withdrawn the rule in order to explore alternative policy actions through re-engagement with stakeholders (United States Department of Agriculture [USDA], 2017b). Uncertainties with rulemaking has led USDA to clarify is current position with respect to genome editing for plant improvement as consistent with the planned updates in regulatory oversight (United States Department of Agriculture [USDA], 2018b).

In concert with the EOP effort to rethink the Coordinated Framework, the FDA requested public comment as to genome edited plant varieties used for food and feed (United States Food and Drug Administration [FDA], 2017). At the time of the request, FDA had “not completed a voluntary food safety consultation on food derived from a plant produced using genome editing” (US Food and Drug Administration [FDA], 2018b). In requesting comments, the FDA is seeking to determine if foods derived from genome edited plants represent “categories of plant varieties” different from plants developed using traditional plant breeding, and if these differences are likely to change food safety risks for human and animal foods.

Governance outside the bounds of the Coordinated Framework is evidenced in local initiatives by Institutional Biosafety Committees (IBC), which have lead researchers at some institutions to self-regulate their design of genome editing research to avoid inadvertent gene drive development (Wolt, 2017). Proactive efforts at the local level have preceded more formalized efforts by NIH to evaluate “the current biosafety oversight framework, and discuss the future direction of biosafety oversight in light of the emergence of new technologies in the life sciences and the evolution in our understanding of risk and safety”^1^. In addition, recent NASEM guidance on gene drive research (a special application of genome editing) has outlined a stepwise approach toward development and deployment of the technology which engages the wider public in the decision-making process at each stage of activity (NASEM, 2016a).

## Administrative Jurisprudence and Regulatory Rulemaking

### Administrative Law and Jurisprudence

The Coordinated Framework for Biotechnology draws on statutes and statutory language that predates and does not anticipate the emergence of bioengineering processes for crop improvement. As such it stands as a particularly strong example of “lawmaking by administrative institutions” (Stack, 2015), which reflects the ascendance of bureaucracy as the center for regulatory policy making within the federal government (Strauss, 1984). Within this context, rule of law considerations are especially important to the appropriate conduct of administrative law (Waldron, 2011; Stack, 2015). Proceeding from the work of Strauss (1984) defining the administrative structure of government, Stack (2015) identifies five central standards that must be met for administrative decisions to be legitimate: such decisions must be properly *authorized*, must meet requirements of public *notice*, must be *justifiable* to those to whom they apply, must be *coherent* with settled law, and must meet standards of *procedural fairness* that involve recognition of extant rights and duties.

Given that until recently administrative law doctrines have not been extensively considered, traditional jurisprudence provides a bridge for understanding administrative law rules of governance in terms of how policy making for genome edited crops has emerged in the United States.

### Discretion in Traditional Jurisprudence

Regulatory agencies are created by, defined by, and circumscribed by the statutes they are empowered to administer (Office of the Federal Register, 2011). But because statutes typically require clarification and interpretation, they cannot be administered directly. Administration of statutory mandates often requires the creation of rules and guidelines that facilitate the implementation of statutory directives. The agencies that administer the US Coordinated Framework for Biotechnology have significant discretion in the articulation of rules and guidelines. This discretion is circumscribed by statute, by precedent, and by requirements in the Administrative Procedures Act which mandate transparency, public disclosure, and opportunities for public comment and which specify judicial overview for all regulatory actions (Code of Federal Regulations [CFR], 2010). In spite of these restrictions, regulatory discretion gives agencies considerable power to structure the regulatory environment. This power can be exercised in ways that promote a variety of different goals: regulatory decisions might facilitate the adoption of new technologies, respond to the preferences or the interests of constituents, protect the environment, and protect against human, animal or environmental harms. These various goals are not always coincident. For instance, consumers might prefer strict protections, but regulation designed to conform to consumer preferences might retard adoption of new technology. Regulations designed to protect human health or the environment are sometimes perceived by producers as inappropriate or excessive restrictions of their freedom to operate. Regulatory rulemaking, therefore, involves the evaluation of trade-offs among stakeholders whose interests are frequently not aligned with one another. Therefore, the ways agencies of government exercise discretion in rulemaking has significant implications for governance.

Traditional scholarship in jurisprudence has focused primarily on issues of legal interpretation that face judges, and has given comparatively less consideration to the very similar problems faced by regulatory rule and decision making. This is a significant oversight, in part because regulatory decision making is enormously important with practical consequences for policy, but also because similar interpretive issues arise in the contexts of regulatory rulemaking and judicial decision making. In both contexts, interpretive decisions are constrained by statute and precedent, but decision making involves a significant degree of discretion on the part of judges and administrators. Just as judges must give weight, via the principle of *stare decisis*, to the decisions of prior courts, regulatory agencies typically give significant weight to the status-quo rules that were put in place by prior administrators. In both contexts, there is an ultimate authority, with the legal power to specify which interpretations are appropriate and legitimate, and which should be set aside. In the courts, the final legal authority is the United States Supreme Court, which is charged to interpret the law and to give authoritative statements defining what the law *is* on any particular matter. In the case of regulatory agencies, the President of the United States, as head of the executive branch of government, has final authority to review and approve proposed administrative rules, often with the help and advice of the Office of Information and Regulatory Affairs (OIRA). Challenges to the interpretation and execution of administrative rules, however, take place within the judicial system.

The rulemaking discretion exercised by regulatory agencies is similar, in important respects, to interpretive discretion exercised by judges. Judges have significant discretion when deciding cases, but like regulatory agencies, their discretion is constrained by statute and by precedent. Positivist, naturalist and pragmatist jurisprudential theories may be understood as different accounts of the way discretion should be exercised by judges. These theories also have important implications, *mutatis mutandis*, for the way discretion should be exercised by regulatory agencies implementing the Coordinated Framework.

#### Legal Positivism

Legal positivists hold that judges may only refer to valid legal rules when deciding cases. Contemporary positivists (Raz, 1970; Hart et al., 2012) hold more generally that a rule’s status as a valid law depends on its institutional pedigree. Any rule that passes through the proper validating process becomes a valid law, and only valid laws may be referred to by judges. Defenders of positivism often note that the theory tightly restricts the range of judicial discretion, increasing the significance of legislative action. Positivists hold that the content of law should properly be the province of democratically elected legislators, not unelected judges or bureaucrats. Accordingly, positivist jurisprudence offers a tightly constrained view of decision making and discretion on the part of the decision maker.

Positivists need not be *originalists*. Originalism is a theory of constitutional interpretation that holds that the constitution – and by extension, laws – should be interpreted in light of the original meanings of the words and concepts employed (Scalia and Gardiner, 2012). One might characterize this view as a claim that in the interpretation of legal texts, judges and administrators should exercise their discretion by searching for the interpretation that best fits with the meanings the words employed had at the time when the law or legal instrument was validated as law. There may be important implications of this view for determining whether genome edited crops and derived foods count as “genetically engineered” or “bioengineered” for the purposes of regulation under the Coordinated Framework (see discussion, following).

Regulators administering the Coordinated Framework for Biotechnology are tasked to decide whether genome edited crops count, under regulatory rules, as GE even when they do not contain rDNA. Their decision is constrained by law and by institutional guidelines, but within these constraints there is considerable discretion to evaluate alternative reasons and to exercise judgment in selecting among them. Vagueness and ambiguity in statutory language challenge regulators who must decide how agencies should treat genome edited crops and derived foods.

According to Hart et al. (2012), positivist jurisprudence suggests vague and ambiguous legal concepts have a core area of application, as well as more marginal or questionable areas of application. The concepts we use to describe GE and genome edited organisms present such a problem. Depending on how they are conceptualized and defined, genome edited organisms could lie in the core or the penumbra of the legal concepts that would make these organisms available for regulatory oversight, or they could be outside the bounds for such oversight. The basis for making such a decision lies outside the realm of legal positivism. While Hart et al. (2012) eloquently described the structure of vague legal concepts, and provided an articulate account of the scope of interpretive analysis, Hart did not develop a positivist theory of interpretive meaning that would be of practical value to regulatory rulemakers.

Consider, for example, the very practical question whether the National Bioengineered Food Disclosure Standard of 2016 (Public Law 114-216; United States Department of Agriculture [USDA], 2018a) applies to labeling foods that are created using genome editing technologies. Section 291 of the standard defines “bioengineering” as follows:

SEC. 291. DEFINITIONS. “In this subtitle: “(1) BIOENGINEERING.—The term ‘bioengineering,’ and any similar term as determined by the Secretary, with respect to a food, refers to a food–” (A) that contains genetic material that has been modified through *in vitro* deoxyribonucleic acid (DNA) techniques; and “(B) for which the modification could not otherwise be obtained through conventional breeding or found in nature.

This standard is separate from the statutes under which the Coordinated Framework operates, but the definition of bioengineering critically intersects with “products of biotechnology” as defined under the revision of the Coordinated Framework for Biotechnology (Executive Office of the President [EOP], 2017) and its proposed implementation in revised USDA rulemaking (United States Department of Agriculture [USDA], 2017b).

While positivism as such does not provide an answer to this quandary, neither does a positivist theory of jurisprudence rule out all reasonable standards that might provide an answer. But some positivists, including Hart, have argued that where available legal materials run out, those tasked to interpret the law do not have discretion to decide based on reasons that positivists consider to be *external* to law. Where legislative rules *expressly* assign discretion to regulatory agencies, the powers created by statutory authority flow directly from a valid law. In this case, the standard expressly assigns the Secretary of Agriculture the power to extend the statutory definition of “bioengineering” to “any similar term,” but does not expressly grant broad discretion concerning the interpretation of statutory language. While its authors obviously tried to be clear, the National Bioengineered Food Disclosure Standard of 2016 does not interpret itself, and its language does not expressly answer whether foods derived through genome editing are products of bioengineering, under the given statutory definition. Furthermore, as a matter of administrative law, it is not clear whether there is coherence in the language and intent of this labeling standard and the bioengineering products addressed through the revised Coordinated Framework.

#### Naturalism and Pragmatism in Traditional Jurisprudence and in Regulatory Rulemaking

Naturalist and pragmatist theories of jurisprudence and statutory interpretation offer a slightly more expansive view of judicial and regulatory discretion. Advocates of jurisprudential naturalism (Dworkin, 1977, 1986, 1996, 2011; Barber and Fleming, 2007) argue, in the context of decision making, for discretion in ways that make the law *best.* Given alternative available interpretations of statutory language, argues Dworkin (1986), decision makers should ask which interpretation most effectively protects rights, promotes well-being, and advances public values embodied in the law and the constitution. According to Dworkin, judges have a degree of discretion in selecting among alternative interpretations, but their discretion is not absolute since they could make better or worse interpretive decisions. While naturalists urge that there are strict boundaries that limit the discretion of judges and others who are tasked to interpret the law, some legal pragmatists (Posner, 1999, 2008, 2013) have argued that such limits are mere rhetoric. According to Posner, judges and other decision makers should appeal broadly to diverse sources of information, from social sciences to economics to the “hard” sciences, to ensure that their decisions will be informed by the best possible understanding of issues surrounding the legal decision in question. As we have discussed, this is consistent with the broad stated intent of Congress with regard to statutes and regulations, but has conflicted with interpretation of statutes such as NEPA and the Endangered Species Act by regulators working under the Coordinated Framework.

##### Naturalist jurisprudence

So-called “naturalism” in jurisprudence is most strongly associated with the work of Dworkin (1977, 1986, 2011) and others (notably Barber and Fleming, 2007). It might seem obvious to say that judges and administrators should select the interpretation that makes the law *best*, but this naturalist view has sometimes been viewed to be at odds with the positivist insistence that the interpreters of law may only appeal to sources *within law* in support of their judgments. The appeal to “what is best” has sometimes been seen as a way to substitute private value judgments for what would otherwise be a more objective legal standard.

Dworkin is careful to note the limits of interpretive discretion and identifies various types of discretion. A decision maker may have “ultimate” discretion when there is no higher decision-making authority who can overrule the decision made and where there is no further appeal. A different kind of discretion applies when application or execution of rules or orders requires the exercise of judgment. In that case, officials who interpret and apply rules may have a range of alternatives available, and within that range will not be bound by standards set by some higher legal authority (Dworkin, 1967). Interpretative and rulemaking discretion that is not *ultimate* may be stronger or weaker, depending on context and institutional circumstances. In administrative law, the *authorization* of regulatory rules will depend, in part, on whether rulemakers act within the bounds of discretion permitted by governing rules and institutional powers. Regulatory officials are vested with the authority to use limited discretionary judgment in the interpretation and execution of statutory law (Stack, 2015).

Within US law, the US Supreme Court has ultimate interpretive discretion, since there is no higher appeal within the structure or extant law. In regulatory rulemaking, it is the President who has ultimate authority over the processes by which regulatory agencies make and apply rules, but this does not represent ultimate discretion because the courts determine whether the processes are conducted consistent with existing law (Code of Federal Regulations [CFR], 2012). In these cases, the legislature can provide a check on the power of the courts or the President by passing legislation that rebuts an unwelcome decision. The kind of discretion can we apply to regulatory agencies tasked to administer the Coordinated Framework for Biotechnology, when they articulate rules that interpret extant regulatory law, is not so clearly defined. These regulators do not have *strong* discretion: their decisions can be better or worse in a variety of different ways. Nor do they have *ultimate* discretion, since there is a higher authority – the President or courts – who may overrule their decisions. Arguably, the discretion of regulatory agencies is similar to that of lower-court judges. Regulatory agencies are responsible to interpret and execute regulatory law. The process requires oversight, public input and appropriate consultation with subject-matter experts. Regulatory rulemaking is not a democratic process, but administrative rulemakers are required to hold public hearings so that the public can exercise its right to influence the process. Presumably this requirement of public input is intended to take public input into account, but the regulators are not bound to do what the public wants. Other legislative and institutional constraints that apply to the rulemaking process are similar: they provide a significant range of choice, within specified constraints. Legal naturalism recommends that regulators should consider the scope of discretion and select among the options that lie within that scope. Among the available options, they should select the one that is best.

Critics of natural law jurisprudence worry that natural law theory may be undemocratic, and that it may give decision makers license to substitute their own personal moral values for the legal rules that should more appropriately constrain their choices. Its defenders, however, urge that naturalism incorporates the best features of positivism without adopting its excessive constraints.

##### Legal pragmatism

Legal pragmatism is a family of loosely related legal theories. For the purposes of this discussion, the term will be associated with the work of Posner where “legal pragmatism” is the idea that legal interpretation is a practical human activity where interpretive practice should not be bound by absolute principles such as “moral, legal, and political theory when offered to guide legal and other official decision making,” (Posner, 2003, p. 3).

Pragmatism as a method leads judges and regulatory rulemakers to recognize decisions may have unexpected consequences which inform later decisions with recognition of prior error and success. Whereas judicial commitment to principles of interpretation may lead decision makers to ignore important data that should properly influence the actions, pragmatism in principle applies no limitation on what kinds of considerations may appropriately provide insight in making decisions, such as recent findings of social and physical sciences or the effect different rulings would have on public opinion. Pragmatists place no in-principle restrictions on the scope of the discretion available to decision makers charged to interpret law and come to a ruling in hard cases. Critics of this view worry, predictably, that it grants too much discretion and too much power to judges and other interpreters of law.

### Challenges of Governance for Products of Biotechnology

The emergence of genome editing as a promising tool for crop improvement has wide ranging implications not only for regulatory consideration of genome edited crops themselves but also to future innovations from the rapidly advancing field of bioengineering. The US Coordinated Framework for Biotechnology has undergone considerable change through time in an attempt to be responsive to the changing nature and understanding of bioengineered plants. While the overall conduct of these regulatory changes show adherence to administrative standards of authority, notice and justification, they may be faulted in terms of procedural fairness (where judicial decisions have compelled a more widely directed consideration of impacts of biotechnology) and, in particular, coherence.

Interpretive rulemaking requires rules that are coherent – that is, they should be consistent with and supported by the underlying legal materials, and should be appropriately linked to other relevantly similar policies. Coherence is not mere consistency; unrelated statements are consistent with one another, but do not form a coherent whole. The coherence of regulatory policy requires in addition that there should be inferential relationships among the different elements of regulatory law – that rulings should be derivable from underlying materials, or (more minimally) that they should constitute a reasonable interpretation of underlying and surrounding elements of the legal framework. Overtime the Coordinated Framework has exhibited a shifting definition of what is the subject of regulatory concern – rDNA, GE organisms, or products of biotechnology. Such a lack of coherence is unavoidable as long as the focus remains on technological processes rather than on the products themselves. A lack of coherence through time elicits uncertainty on the part of scientists, developers and the public as well as for regulators themselves. The pending implementation of labeling under the National Bioengineered Food Disclosure Standard portends further problems with administrative coherence, since labeling of “bioengineered” foods and its alignment with the Coordinated Framework leaves uncertainty as to which “products of biotechnology” (actually a process consideration as defined under the Coordinated Framework) will be labeled as bioengineered.

As an instance of administrative law, the administrative jurisprudence of the Coordinated Framework – as informed by traditional theories of jurisprudence – will face challenges for its continuance from both inside and outside of government given the accelerating novelty in approaches whereby bioengineering of organisms may be accomplished. In the sequence of views considered here, positivists and originalists would be the least amenable to interpretive discretion in rulemaking under the Coordinated Framework, since they would rely only on existing legal material which is lacking in cases where new technology may have no precedent in law. Legal naturalists, however, argue that in cases involving public dispute, failure to decide serves as a legal precedent, and urge that the discretion available must include the ability to make decisions consistent with law, but also sanctioned by legal principles that guide law. Those who interpret the existing state of policy with respect to genome edited crops as a nondecision may fall into this category. The work of legal pragmatists, like that of positivists and naturalists, has mostly focused on the interpretive role of judges, but the view has natural application to the problems of legal interpretation faced by regulatory rulemakers, including the implications of extant regulation of GE crops to proposed regulation of genome edited crops and derived foods. Legal pragmatism would argue for regulatory principles that are better gauged toward current day scientific and societal understanding of the risk and benefits of genome edited crops.

## Summary

Our purpose in this discussion has been to elaborate how governance within the US legal framework is influencing decisions regarding the regulation of genome edited crops. We do not defend or justify the US regulatory system or suggest any given theory of jurisprudence which is preferable for administration of the Coordinated Framework for Biotechnology. Such considerations would require much more serious examination of the norms that constitute the basis of the US regulatory system. However, this analysis of the regulatory framework for biotechnology in the US should provide an explanation of the circumstances in law that have led US regulatory agencies, including the USDA, to their current positions for imposing new rules for crops and derived foods developed through genome editing.

## Author Contributions

The authors contributed equally to ideation and development of this contribution. JW developed the historical review of the Coordinated Framework and discussion of its implications toward genome edited crops. CW developed the jurisprudence content and its use for interpreting regulatory decisions with respect to genome edited crops.

## Conflict of Interest Statement

The authors declare that the research was conducted in the absence of any commercial or financial relationships that could be construed as a potential conflict of interest.

## Footnotes

1 https://osp.od.nih.gov/event/nih-guidelines-honoring-the-past-charting-the-future/?instance_id=39

## Abbreviations

DNA: deoxyribonucleic acid
EA: environmental assessment
EIA: environmental impact assessment
EIS: environmental impact statement
EPA: United States Environmental Protection Agency
ESA: Endangered Species Act
EOP: United States Executive Office of the President
FDA: United States Food and Drug Administration
FWS: United States Fish and Wildlife Service
GE: genetically engineered
NASEM: United States National Academies of Science, Engineering and Medicine
NEPA: National Environmental Protection Act
NIH: United States National Institutes of Health
NMFS: United States National Marine Fisheries Service
OSTP: United States Office of Science and Technology Policy
PIP: plant incorporated protectant
RAC: Recombinant DNA Advisory Committee
rDNA: recombinant DNA
synDNA: synthetic DNA
USDA: United States Department of Agriculture.
